# Genome Sequences of Two Cyanobacterial Strains, Toxic Green *Microcystis aeruginosa* KW (KCTC 18162P) and Nontoxic Brown *Microcystis* sp. Strain MC19, under Xenic Culture Conditions

**DOI:** 10.1128/genomeA.00378-18

**Published:** 2018-05-03

**Authors:** Haeyoung Jeong, Seong-Jun Chun, Ankita Srivastava, Yingshun Cui, So-Ra Ko, Hee-Mock Oh, Chi-Yong Ahn

**Affiliations:** a Infectious Disease Research Center, Korea Research Institute of Bioscience and Biotechnology (KRIBB), Yuseong-gu, Daejeon, Republic of Korea; b Cell Factory Research Center, Korea Research Institute of Bioscience and Biotechnology (KRIBB), Daejeon, Republic of Korea; c Department of Biosystems and Bioengineering, KRIBB School of Biotechnology, Korea University of Science and Technology (UST), Daejeon, Republic of Korea; d Department of Environmental Biotechnology, KRIBB School of Biotechnology, Korea University of Science and Technology (UST), Daejeon, Republic of Korea

## Abstract

Bloom-forming cyanobacteria pose concerns for the environment and the health of humans and animals by producing toxins and thus lowering water quality. Here, we report near-complete genome sequences of two Microcystis strains under xenic culture conditions, which were originally isolated from two separate freshwater reservoirs from the Republic of Korea.

## GENOME ANNOUNCEMENT

Microcystis spp., representative bloom-forming cyanobacteria, can be divided into toxic and nontoxic strains, depending on the possession of an *mcy* gene cluster. Although most Microcystis strains commonly show a green color, brown Microcystis strains have been infrequently isolated from China, Thailand, and Israel (1, 2) . The toxic green Microcystis sp. strain KW (3) and a nontoxic brown Microcystis strain MC19, were isolated from Wangsong Reservoir and Lake Seo, Republic of Korea, respectively. These two strains with contrasting characteristics were investigated from the genomic perspective.

Cells were grown in BG-11 medium under 50 μmol photons m^-2^ s^-1^ at 25°C. Genomic DNA was extracted using FastDNA Spin kit for soil (MP Biomedicals, Santa Ana, CA, USA). A genomic library was constructed using a TruSeq LT sample prep kit, and 2 × 301-cycle reads, with ∼2.7 Gb per sample, were produced on the Illumina MiSeq platform (Korea Research Institute of Bioscience and Biotechnology [KRIBB], Daejeon, Republic of Korea). Long-read sequences, at about 1 Gb per sample, were produced on the PacBio RS II the platform using P6-C4 chemistry at the National Instrumentation Center for Environmental Management, Seoul National University (Seoul, Republic of Korea). Primary assemblies were chosen on the basis of contig numbers and total length, out of the results from three independent runs of the long-read assemblers Canu version 1.3 (4), Falcon version 0.3 (5), and SMRT Analysis version 2.3 (RS_HGAP_Assembly.2). For strain KW, Falcon yielded the smallest set of 32 contigs; they were polished by two additional rounds of RS_Resequencing.1 under the SMRT Analysis environment. Paired MiSeq reads, after adaptor removal and quality trimming using Trimmomatic version 0.32 (6), were mapped to the contigs, which divided contigs into two groups according to read coverage and length. Completeness was checked by the presence of PhyloSift marker genes (7). The top 10 contigs (5.8 Mb), after independent joining using CLC Genomics Workbench, PB-Jelly (8), and RS_AHA_Scaffolding in SMRT Analysis, were manually integrated into 6 contigs. Meanwhile, long-read assemblies using strain MC19 produced a 5.0-Mb contig (the largest) consistently with smaller ones using any of the three assemblers. The largest of the 151 contigs obtained from Canu was circularized by mapping the corrected reads in CLC Genomics Workbench, and RS_AHA_Scaffolding was performed again. The final contigs from both strains were further polished using the Pilon version 1.20 (9).

Genome annotation was carried out using NCBI Prokaryotic Genome Annotation Pipeline (https://www.ncbi.nlm.nih.gov/genome/annotation_prok/). BLASTX analysis of coding sequences from KW against the RefSeq genome database using Blast2GO revealed that 94.7% of the best hits were related to the genus Microcystis, which implies that the initial selection of primary contigs was successful. Contig1t (102,002 bp) was assigned a putative conjugative plasmid due to its higher read coverage and GC content than those of the rest of the assembly and the presence of plasmid-carried genes. Genome-wide average nucleotide identity (gANI) analysis (10) with the type strain NIES-843 classified only strain KW into Microcystis aeruginosa species unequivocally. Among the available genome sequences from Microcystis strains, T1-4 (GenBank accession number CAIP00000000) was most closely related to MC19 (99.2% gANI).

### Accession number(s).

The genome sequences of the two cyanobacterial strains have been deposited in DDBJ/ENA/GenBank under the accession numbers MVGR00000000 (KW) and CP020664 (MC19).
